# Rice Grain Detection and Counting Method Based on TCLE–YOLO Model

**DOI:** 10.3390/s23229129

**Published:** 2023-11-12

**Authors:** Yu Zou, Zefeng Tian, Jiawen Cao, Yi Ren, Yaping Zhang, Lu Liu, Peijiang Zhang, Jinlong Ni

**Affiliations:** 1 Rice Research Institute, Anhui Academy of Agricultural Sciences, Hefei 230031, China; zouyu@aaas.org.cn; 2 College of Engineering, Anhui Agricultural University, Hefei 230036, China; 21114165@stu.ahau.edu.cn (Z.T.); jiawencao0916@ahau.edu.cn (J.C.); 3 College of Agriculture, Anhui Science and Technology University, Chuzhou 239000, China; yjs2022230@ahstu.edu.cn; 4 Hefei Institute of Technology Innovation Engineering, Chinese Academy of Sciences, Hefei 230094, China; 17861856476@stu.ahau.edu.cn (Y.Z.); vliulu@ahau.edu.cn (L.L.)

**Keywords:** rice grain detection and counting, YOLOv5, coordinate attention module, transform

## Abstract

Thousand-grain weight is the main parameter for accurately estimating rice yields, and it is an important indicator for variety breeding and cultivation management. The accurate detection and counting of rice grains is an important prerequisite for thousand-grain weight measurements. However, because rice grains are small targets with high overall similarity and different degrees of adhesion, there are still considerable challenges preventing the accurate detection and counting of rice grains during thousand-grain weight measurements. A deep learning model based on a transformer encoder and coordinate attention module was, therefore, designed for detecting and counting rice grains, and named TCLE-YOLO in which YOLOv5 was used as the backbone network. Specifically, to improve the feature representation of the model for small target regions, a coordinate attention (CA) module was introduced into the backbone module of YOLOv5. In addition, another detection head for small targets was designed based on a low-level, high-resolution feature map, and the transformer encoder was applied to the neck module to expand the receptive field of the network and enhance the extraction of key feature of detected targets. This enabled our additional detection head to be more sensitive to rice grains, especially heavily adhesive grains. Finally, EIoU loss was used to further improve accuracy. The experimental results show that, when applied to the self-built rice grain dataset, the precision, recall, and mAP@0.5 of the TCLE–YOLO model were 99.20%, 99.10%, and 99.20%, respectively. Compared with several state-of-the-art models, the proposed TCLE–YOLO model achieves better detection performance. In summary, the rice grain detection method built in this study is suitable for rice grain recognition and counting, and it can provide guidance for accurate thousand-grain weight measurements and the effective evaluation of rice breeding.

## 1. Introduction

Rice is one of the most important cereal crops in the world. Improvements in rice yields and quality can help meet the growing demand for food throughout the world. Rice yield information plays a critical role in the management of rice production, guiding agricultural planting practices and assisting breeding decisions. Such practices and decisions are related to grain weight, which is usually represented as thousand-grain weight [1,2]. Thousand-grain weight is an important indicator used to evaluate variety breeding, and it is not only an important parameter for the effective evaluation of grain yields and milling quality, but it also has an impact on seedling vigor and growth, both of which indirectly affect yield [3]. As a result, thousand-grain weight is frequently used in rice breeding research as a measurement indicator. The key to measuring the thousand-grain weight of rice grains is to accurately count the rice grains.

Traditionally, thousand-grain weight measurements were mainly obtained by counting the grains manually, which is time-consuming, labor-intensive, and highly prone to human error, especially in long-term observation and counting processes. It is, therefore, important and urgent to realize the automatic detection and counting of rice grains so as to fulfill the requirement of the rapid and accurate counting of rice grains on a large scale. Recently, along with advancements in machine vision systems, a less costly and more easily implemented method of crop detection and counting has been developed in the form of image processing technology [4]. Usually, the visible features of a crop, including color, size, shape, and texture, are extracted through image analysis and processing, following which the crop can be segmented from the background based on one or a combination of the above characteristics and counted [5,6,7]. The characteristics described above, such as color and texture, are low-level features obtained using artificial feature extraction methods; these are highly accurate for detecting and counting crops in a sparse state. However, rice grains are small targets with a high overall similarity in color and morphological characteristics, but low resolution. They also possess different degrees of adhesion which, during thousand-grain weight measurements, complicates the task of distinguishing rice grains and affects counting accuracy. Hence, it is difficult to obtain accurate and stable features using image-processing methods involving manually designing features according to changes in the target; such methods are of limited value in the rapid and accurate detection and counting of rice grains.

Compared with the image processing methods outlined above, object detection methods based on deep learning enable accurate and reliable crop counting due to their strong feature extraction ability and autonomous learning ability [8,9,10]. Khaki et al. [11] presented a sliding window approach based on a CNN classifier for the detection and counting of corn cob grains; they achieved an RMSE of 8.16% of the average number of grains in a grain-counting task. Gong et al. [12] designed a fully convolutional network to achieve the goal of detecting grains within a panicle, realizing an accuracy of 95%. Tan et al. [13] applied YOLOv4 to detect cotton seedlings in individual frames and adopted an optical flow-based tracking method to estimate camera motions and obtain the number of cotton seedlings. Lyu et al. [14] detected and counted green citrus fruits in orchards based on an improved YOLOv5 by embedding a convolutional block attention module and a detection layer, and their results showed that, for green citrus fruits, the mAP@0.5 of the proposed model was 98.23% and the recall was 97.66%. Rong et al. [15] proposed a tomato cluster detection model based on an improved YOLOv5-4D that fused RGB images and depth images as input, and they achieved an accuracy of 97.9% and an mAP@0.5:0.95 of 0.748.

These deep-learning-based object detection algorithms can provide an accurate and efficient way to count targets. However, due to the small size of rice grains, their highly similar phenotypes, and the low resolution of a single grain, image feature information containing a sufficient degree of discrimination between adhesive grains only usually exists in a very small and local area and is not easily learned by a network. As a result, the detector may miss some of the grains, which affects counting accuracy, and, in turn, affects the accuracy of yield estimations. Further, it is well known that thousand-grain weight is also closely associated with grain-size traits, such as grain length, grain width, grain thickness, and the kernel length/width ratio [16]. The misidentification and miscounting of rice grains can affect the accuracy of grain-size traits automatically obtained based on image technology, as well as the effective evaluation of cultivation measures, seed phenotypic analysis, variety breeding, rice grain sorting, etc. Therefore, the key to the detection and counting of rice grains is to effectively extract and utilize useful feature information from these local regions of rice grains based on deep learning, especially in the effective detection of heavily adhesive rice grains, which are difficult to distinguish.

To the best of our knowledge, the attention module resembles the human visual attention mechanism in that it pays attention to part of the regional information, enabling the realization of the task while filtering out the secondary data. This improves the effectiveness of the model when processing information, and it can be used to enhance the feature perception of network models, which can be also applied to the identification of small targets that are occluded or which adhere to each other [17]. Peng et al. [18] designed a soybean aphid identification model based on a CNN with an attention mechanism, and this produced higher accuracy. Zhang et al. [19] proposed a method for imperfect wheat-grain recognition combined with an attention mechanism and a residual network (ResNet). Their results showed that the introduction of the attention mechanism improved recognition accuracy when classifying perfect wheat grains and five different types of imperfect wheat grains. We et al. [20] noted that algorithm detection accuracy could be improved by using an attention mechanism; they further noted that among the various improvements that have been made to object detection models, embedding an attention module into the model was one of the most effective.

The shortcomings of current grain detection and counting methods based on image analysis technologies, as well as the characteristics of adhesive rice grains, which render them difficult to distinguish, necessitate the design of a model based on deep learning which would fulfill the following requirements: (1) the model should have good feature recognition ability and should electively focus on the effective features of target regions, ensuring the robustness of the model for adhesion target region recognition; (2) the model should differentiate between adhesive rice grains and automatically locate target regions with different degrees of adhesion so as to accurately distinguish between the target regions with different degrees of adhesion; and (3) the model should automatically locate small targets with highly similar phenotypes to ensure that the detector can detect rice grains more sensitively.

Based on the above research and analysis, this study will attempt to design a detection and counting method for rice grains by combining an attention mechanism with YOLOv5, hereafter named TCLE–YOLO. Firstly, to address the difficulty of distinguishing adhesive rice grains, we introduce the coordinate attention (CA) module [21] into the YOLOv5 backbone module; this is intended to improve the ability of the model to focus on small targets, thereby enhancing the feature expression ability of the network. Secondly, an additional detection head that is more sensitive to small targets is designed based on a low-level, high-resolution feature map generated by the transformer encoder; this is intended to improve the small target detection capability [22]. In addition, the transformer encoder is applied to the neck module to expand the receptive field of the network and make sure the network pays more attention to the effective feature information about the rice grain region. This makes the additional detection head more sensitive to small-sized objects. The remainder of this paper is structured as follows: Section 2 presents the data sources and research methods employed in the development of TCLE–YOLO, Section 3 presents the experimental results and discussions, and the conclusions are presented in Section 4.

## 2. Materials and Methods

### 2.1. Materials

#### 2.1.1. Image Acquisition and Preprocessing

**Figure 1:**
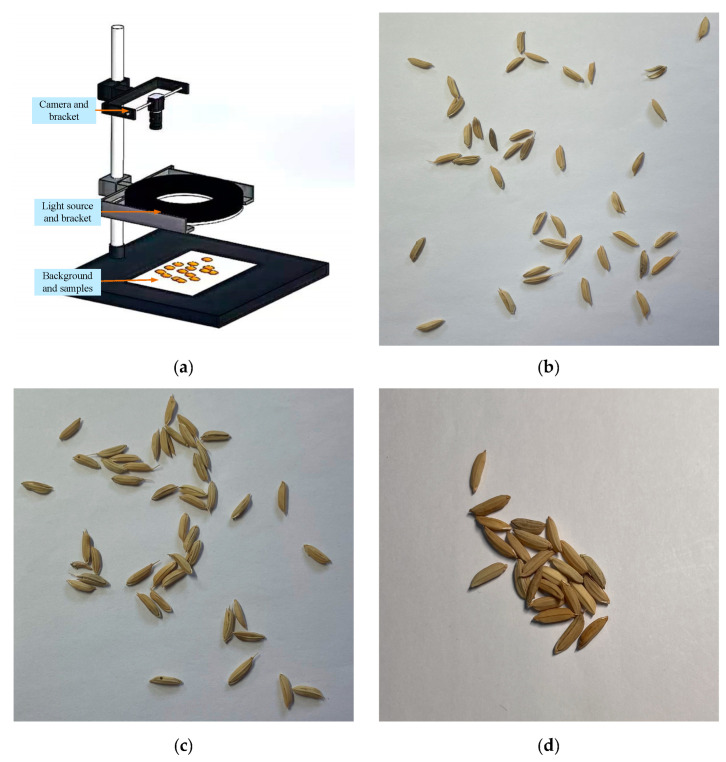
Image acquisition platform and sample images. (**a**) Mobile phone camera working diagram; (**b**) sample image of mildly adhered rice grains; (**c**) sample image of moderately adhered rice grains; (**d**) sample image of severely adhered rice grains.

The experimental materials used in this study were produced at the Rice Research Institute, Anhui Academy of Agricultural Sciences. The rice grains were randomly laid on a white background board during capture. A camera was then used to capture images of the rice grains, and these were the original images. As Figure 1 shows, a mobile phone camera was installed on a visual platform to capture images of the rice grains. The camera holder was adjusted up and down to enable the camera bracket to simulate different distances. In this study, the mobile phone camera was used to capture images of rice grains of different degrees of adhesion at capture distances of 8 cm, 15 cm, and 20 cm away from the white background board. In accordance with [23], a local area of an image containing 2~10 grain adhesions was defined as showing ‘mild adhesion’, while a local area containing 10~20 grains was defined as showing ‘medium adhesion’, and a local area containing more than 20 grains was defined as showing ‘severe adhesion’. In total, 1000 rice grain images were taken at a resolution of 3024 × 4032, including 250 images of mildly adhered rice grains, 400 images of moderately adhered rice grains, and 350 images of severely adhered rice grains.

Several sample images of rice grains of different degrees of adhesion are shown in Figure 1. The acquired images were in RGB format. The resolution of the original images was much higher than the input image size for YOLOv5. There was a possibility that the high resolution of the original images would increase the difficulty of the network training and lead to the overuse of the GPU memory, and consequently to training failure. To avoid this situation, the acquired rice grain images were scaled to reduce the resolution to 640 × 640.

#### 2.1.2. Image Augmentation and Annotation

**Figure 2:**
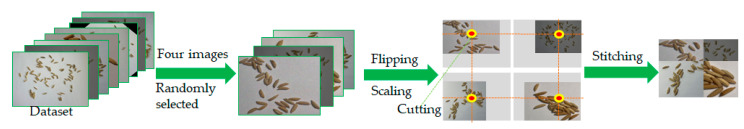
Schematic diagram of mosaic data augmentation.

**Figure 3:**
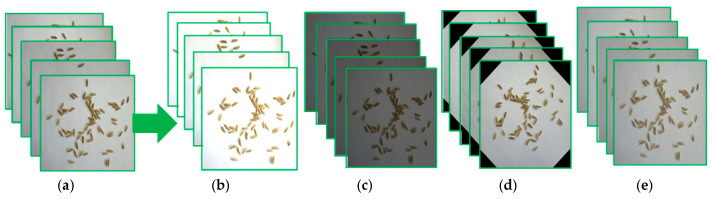
Image augmentation and sample images. (**a**) Original sample images of rice grains; (**b**) sample images with brightness enhancement; (**c**) sample images with reduced brightness; (**d**) rotated sample images; (**e**) sample images with added noise.

It is generally recognized that training a deep neural network requires a large number of images, and that using data augmentation can increase the amount of image data and significantly improve the performance of the model [24]. Mosaic data augmentation [25] can randomly select four images which are then randomly scaled, flipped, and arranged to achieve re-switching and data augmentation, as is shown in Figure 2. In addition, common data augmentation methods including brightness conversion, multi-angle image rotation, and adding noise were also applied to the original images to greatly enrich the number of samples for the detection and counting of the rice grains. After data augmentation, the number of samples in the dataset was expanded to 6000. Part of a rice grain image after data augmentation is shown in Figure 3.

**Figure 4:**
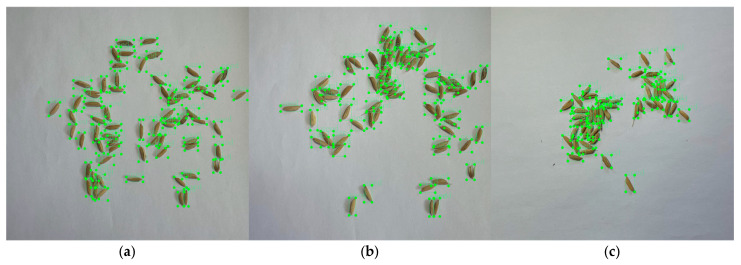
Sample annotated images. (**a**) Sample annotated image of mildly adhered rice grains; (**b**) sample annotated image of moderately adhered rice grains; (**c**) sample annotated image of severely adhered rice grains.

The ground truth (GT) images of the object detection model were bounding box labeled. To obtain the labeled samples, LabelImg annotation software was used to manually label the images in the dataset. As Figure 4 shows, the areas containing the rice grains were marked as green bounding boxes. The annotation results were stored in an xml file and used to train the model with the training dataset and to calculate the performance of the model with the validation and test datasets. The RGB rice grain images were randomly allocated to the training and validation datasets in a ratio of 7:2, and the remaining images were used as a test set to evaluate the detection performance and robustness of the proposed model.

### 2.2. Model for Detecting and Counting Rice Grains

YOLO (you only look once), a representative framework for single-stage detection, is a high-performance general-purpose target-detection model which has spawned many versions. Among these, YOLOv5 has a smaller and more flexible structure and faster image inference. It has been employed to develop real-time object detection models [26]. YOLOv5 consists of a backbone module, a neck module, and a head module, of which the backbone module is the basis of the YOLOv5 model, extracting the features of the input images. The neck module, including several bottom-up paths and top-down paths, mainly up-samples the features extracted by the backbone module and fuses the feature information from different network layers. The detection head module mainly predicts image features and is responsible for predicting the category, location, confidence, and other information in a target detection task. YOLOv5 was used as the backbone framework in the detection and counting model outlined in this paper. However, as has already been recognized, the feature pyramid network with multiple convolutions used in YOLOv5 can cause some target feature information to be easily lost during training, leading to poor detection of small targets [27]. Moreover, rice grains are small, low-resolution targets with high overall similarity in terms of color and morphological characteristics, and adhesion and occlusion occurs between rice grains. This severely limits the accuracy with which rice grains can be identified and counted. When detecting small targets, high-resolution representations are required, and the representation of these objects may not even be available in the deep-layer features, making small target detection difficult.

**Figure 5:**
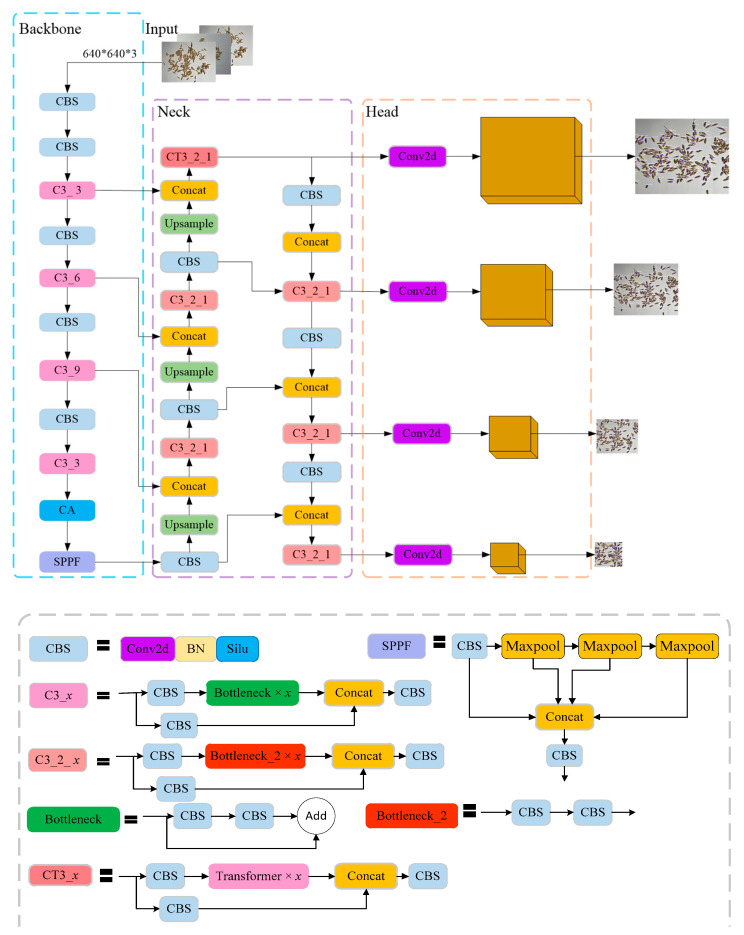
Structure of the improved YOLOv5 model and composition diagram of each module.

Because of this, and to improve the performance of the model and its adaptability to adhesion targets for the detection and counting of rice grains, we embedded a coordinate attention (CA) module into the backbone module of YOLOv5. We also introduced a transformer encoder to the neck module and added a detection head that is sensitive to small-sized objects. Figure 5 illustrates the architecture of our model for detecting rice grains. As is shown in the figure, the coordinate attention module was positioned before the SPFF block of the backbone module to enhance its ability to extract detailed information about small targets. The transformer encoder was introduced to the level-layer features to enhance the perception field and make the model pay more attention to the effective feature information of the rice grain region. The low-level, high-resolution feature map output from the transformer encoder was then sent to the detection head that was added to detect small-sized targets.

#### 2.2.1. Improved Backbone Module

The backbone module of the YOLOv5 model is mainly used to extract image features, and it consists of repeated CBS and C3_*x* blocks followed by an SSPF for feature fusion. However, in the backbone module, image features are gradually transformed from shallow to deep, and the size of the corresponding image decreases at the same time. This results in the semantic information of small targets usually being lost during feature transmission with multiple convolutions. Moreover, rice grains are small targets with high overall similarity and different degrees of adhesion, and this must be accounted for during thousand-grain weight measurements. To accurately detect rice grains, especially adhering rice grains, it is necessary for the detection model to extract semantic and location information that is critical to the current task goal. In [28], Li et al. stated that an attention mechanism can make the deep learning network pay different levels of attention to different parts of input images and select feature information that is more critical to the current task, thus significantly improving the performance of the network. Based on the role of the attention mechanism described in [28], we introduced a coordinate attention (CA) module to emphasize meaningful semantic and location features. The CA module was placed before the SSPF block, as is shown in Figure 5.

**Figure 6:**
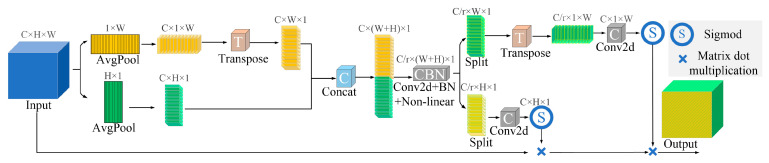
Structure diagram of the coordinate attention module.

The CA module mainly carries out coordinate information embedding and coordinate attention generation. Let the coordinate attention input be a feature map X with dimensions of C × H × W. As is seen in Figure 6, the coordinate information is embedded by encoding each channel on the input along the horizontal and vertical coordinates using two spatial pooling kernels, (H, 1) and (1, W). This produces two direction-aware attention maps—a horizontal direction feature map with dimensions of C × H × 1 and a vertical direction feature map with dimensions of C × 1 × W. In the two attention maps, each element reflects whether or not the interest region exists in the corresponding row and column, and long-range dependencies along one spatial direction with precise positional information can be captured. The two feature maps are then concatenated in the channel dimension and sent to a convolutional transformation to generate an intermediate feature map with dimensions of C × (W + H) × 1. To generate the attention weight maps along the horizontal and vertical directions, the intermediate feature map is split along the spatial dimension into two separate feature maps, and these split feature maps are then transformed by two convolutions. This allows the two transformed feature maps to have the same number of channels as the input X. The maps are subsequently passed through the sigmoid normalization function to obtain a horizontal attention weight map and a vertical attention weight map. Finally, the two attention weight maps are multiplied by the input X to generate a new feature map of the enhanced X. In this way, the CA module encodes the generated feature maps to form a pair of direction-aware and position-sensitive feature maps which can enhance the coordinate information of the rice grains and hence help the detection model to recognize them more accurately.

#### 2.2.2. Design of Multiple Detection Heads

The detection head module is located behind the neck module, and it is mainly responsible for predicting categories and generating bounding boxes based on image features. However, for small targets, feature representation conveys less semantic information, and bounding box positioning is more challenging than it is for larger targets. In addition, in the regression process of the detection head, the regression bounding box is offset by one pixel, and this has a considerable impact on the error of the small target. The deviation of the small target prediction bounding box also affects the network’s target classification result during the classification process of the detection head. As mentioned in [27], detection heads based on convolutional neural networks often analyze deep feature maps with less feature information, and this is not conductive to the detection of small objects. Conversely, low-level features with spatial-rich information have higher resolutions and are, thus, more suitable for detecting small targets. Therefore, in order to solve the problem of the difficulty of detecting small targets, a detection head sensitive to small targets was added. 

As Figure 5 shows, the designed prediction head was generated from the low-level, high-resolution feature map, and it was more sensitive to small targets. Based on YOLOv5, the neck module was inserted between the backbone module and the head module; it was designed to make better use of the features extracted by the backbone and is a key link in the target detection framework. Wu et al. [27] indicated that large receptive fields and high-resolution spatial information are required to correctly detect and localize small targets. In order to obtain a large receptive field in the neck, a transformer encoder block with a self-attention mechanism was embedded into the YOLOv5 C3 block, enabling the generated feature map from the transformer encoder block to be sent to our prediction head. This ensured that the model would be able to identify the characteristics of rice grains of varying degrees of adhesion to achieve complete detection.

**Figure 7:**
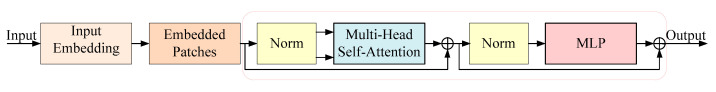
Structure diagram of the transformer encoder block.

The structure of the transformer encoder block is shown in Figure 7. The transformer encoder block consists of two sub-layers. The first sub-layer is the multi-head attention mechanism, in which the feature dimensions are distributed into multiple single-head self-attention mechanisms according to the importance of particular image regions. This enables the network to pay more attention to key information and allows it to match the extracted features to the detected targets. By stitching multiple attention results, contextual semantic information is obtained. This is conducive to mining fine features and enriching the extracted features. The second sub-layer is an MLP, which is a fully connected layer of the feed-forward neural network. It prevents network degradation by transforming features. Both sub-layers use residual connections to connect the fusion features between the two sub-layers. In addition, layer normalization is performed before and after the two sub-layers, which can make the network converge faster and avoid over-fitting. Hence, the transformer encoder is applied before the detection head for small targets; this covers the global information for the image more effectively and enhances the transmission of context information, thus facilitating the better detection of rice grains of varying degrees of adhesion.

#### 2.2.3. Loss Function

The design of the loss function can impact on the performance of a network. The original loss function of YOLOv5 is CIoU loss. If the predicted box is **B** and the ground truth box is $B^{gt}$, CIoU loss is formulated as follows:

(1) $$L_{CI\mathrm{ou}}=1-IoU+\frac{\rho^{2}(b,b^{gt})}{c^{2}}+av$$

where *IoU* is the intersection ratio of **B** and $B^{gt}$; *c* represents the shortest diagonal length of the smallest bounding box covering **B** and $B^{gt}$, and $\rho^{2}(b,b^{gt})$ represents the Euclidean distance between the center points of **B** and $B^{gt}$. *a* and *v* are used to measure the discrepancies of the width-to-height ratios of **B** and $B^{gt}$, which are calculated as follows:

(2) $$v=\frac{4}{\pi^{2}}{\left(\arctan\frac{w^{gt}}{h^{gt}}-\arctan\frac{w}{h}\right)}^{2}$$

(3) $$a=\frac{v}{(1-IoU)+v}$$

where *w* and *h* are the height and width of **B**, respectively, and $h^{gt}$ and $w^{gt}$ are the height and width of $B^{gt}$, respectively.

**Figure 8:**
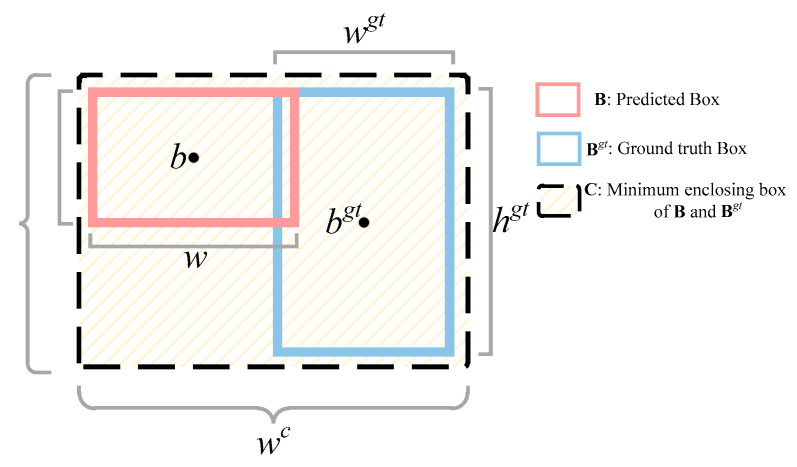
Ground truth box and prediction box.

CIoU loss considers the overlap area, aspect ratio, and central point distance, comprehensively, but it does not consider the difference in width and height between **B** and $B^{gt}$, which leads to a slow convergence speed. Thus, EioU loss was used in this study. As is shown in Figure 8, the blue box is the ground truth box $B^{gt}$, the red box is the prediction box **B**, and the black box is the smallest box covering the ground truth and prediction boxes. Hence, EIoU loss is calculated as follows:

(4) $$\begin{array}{l} L_{EIoU}=L_{IoU}+L_{dis}+L_{asp}\\ \;\;=1-IoU+\frac{\rho^{2}(b,b^{gt})}{{\left(w^{c}\right)}^{2}+{\left(h^{c}\right)}^{2}}+\frac{\rho^{2}(w,w^{gt})}{{\left(w^{c}\right)}^{2}}+\frac{\rho^{2}(h,h^{gt})}{{\left(h^{c}\right)}^{2}} \end{array}$$

where $w^{c}$ and $h^{c}$ are the width and height of the smallest enclosing box covering **B** and $B^{gt}$. As is shown in Equation (4), the loss function is divided into IoU loss, distance loss, and aspect loss, which directly minimizes the difference in width and height between **B** and $B^{gt}$, thus obtaining faster convergence and a better localization result.

## 3. Results and Discussion

### 3.1. Experimental Setup and Evaluation Metrics

The experimental environment was the Windows 10 operating system. The model was implemented using the PyTorch deep learning framework with Torch version 1.13 and CUDA version 11.6. The graphics card used was a NVIDIA GeForce RTX 4080 (2788 San Tomas Expressway, Santa Clara, CA, USA), the CPU was an Intel I7-11700K (2200 Mission College Blvd., Santa Clara, CA, USA), and the memory was a 32GB DDR4 3200. During the training process, the input image resolution was set to 640 × 640, and SGD was used as the optimization function to train the model. The model training epoch was 200, with a batch size of 16 and an initial learning rate of 0.01. The rice grain dataset was used to train and test the model.

To evaluate the detection performance of the model, we adopted precision (*P*), recall (*R*), and mean average precision (*mAP*) as evaluation indicators. These are defined as follows.

(5) $$P=\frac{TP}{TP+FP}$$

(6) $$R=\frac{TP}{TP+FN}$$

(7) $$AP=\int_{0}^{1}P(R)dR$$

(8) $$mAP=\frac{\sum AP}{N}$$

As expressed in Equations (5) and (6), true positive (TP) indicates the number of rice grains detected correctly, and false positive (FP) represents the number of backgrounds incorrectly detected as rice grains. False negative (FN) represents the number of rice grains wrongly detected as background. To obtain TP and FP, the ground truth box and the prediction box identified by the detection model first have to be obtained. The contents of the prediction box include the category, confidence score, and coordinate information. The prediction results are retained and sorted according to the decreasing confidence score when the confidence score is greater than 0.5. The maximum matching IoU values from the prediction box and the ground truth box are then calculated. If they are greater than 0.5 and it is the first match, the result is denoted as TP; otherwise, it is denoted as FP. Therefore, the higher the value of TP, the greater the probability of correct prediction and the better the detection performance of the model; likewise, the more serious the false detection, the worse the performance of the model. Based on this, ‘precision’ refers to the proportion of the total detection results that are correct detection results, and ‘recall’ represents the ratio of the correct detection results to all true results. ‘Mean average precision’ (mAP) represents the average value of each category of AP, and it is defined as the area under the precision–recall curve. It reflects the global detection performance of the model in a more balanced manner. mAP@0.5 signifies a mAP value with an intersection over union (IoU) threshold of 0.5, and mAP@0.5:0.95 indicates the average mAP at different IoU thresholds, from 0.5 to 0.95 in increments of 0.05. In our experiments, the different detection models were trained using the same dataset and experimental settings. After a certain training epoch, the above evaluation metrics for each detection model in the validation set plateaued, indicating that these models had converged on the dataset. The precision, recall, mAP@0.5, and mAP@0.5:0.95 for each trained model were then compared on the test set.

### 3.2. Ablation Experiment

In this paper, ablation experiments were carried out to demonstrate the performance of the different modules of the proposed TCLE–YOLO model, which used YOLOv5 as the backbone network design. Table 1 shows the precision, recall, mAP@0.5, and mAP@0.5:0.95 indicators obtained using the different modules, including the coordinate attention module and the transformer encoder block described above. From the table, it is clear that TCLE–YOLO, which combines a CA module, a transformer encoder block, a small target prediction head, and EIoU loss, achieved the best detection performance for rice grains in terms of the precision, recall, mAP@0.5, and mAP@0.5:0.95 indicators. Compared with YOLOv5, the integrated CA module and the transformer encoder block improved the accuracy from 0.979 to 0.984. Thereafter, the small target prediction head we added to the model improved the accuracy to 0.991. Using the EIoU loss function also improved the accuracy slightly. The final average accuracy of TCLE–YOLO (mAP@0.5) was 0.992, which was 1.74% higher than that of YOLOv5. Moreover, the mAP@0.5:0.95, *P*, and *R* values were higher than those of the original YOLOv5.

**Figure 9:**
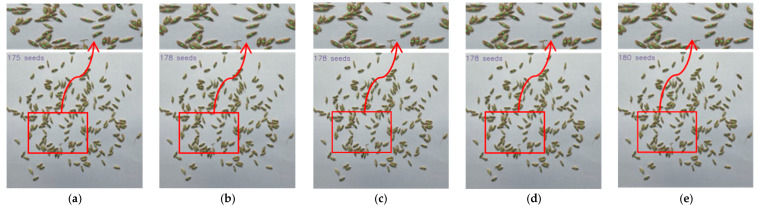
Comparison of detecting and counting results of YOLOv5s with different modules for mildly adhesive rice grains. (**a**) Detecting and counting results of YOLOv5; (**b**) detecting and counting results of YOLOv5 + transformer; (**c**) detecting and counting results of YOLOv5 + transformer + CA module; (**d**) detecting and counting results of YOLOv5 + transformer + CA module + 4 prediction heads; (**e**) detecting and counting results of TCLE–YOLO.

**Figure 10:**
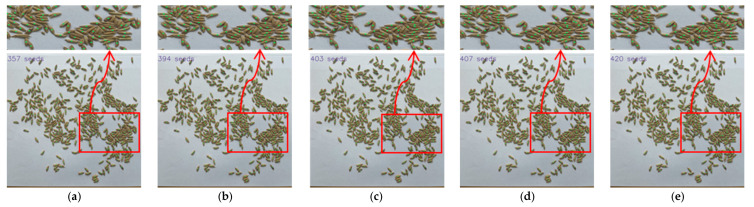
Comparison of detecting and counting results of YOLOv5s with different modules for moderately adhesive rice grains. (**a**) Detecting and counting results of YOLOv5; (**b**) detecting and counting results of YOLOv5 + transformer; (**c**) detecting and counting results of YOLOv5 + transformer + CA module; (**d**) detecting and counting results of YOLOv5 + transformer + CA module + 4 prediction heads; (**e**) detecting and counting results of TCLE–YOLO.

**Figure 11:**
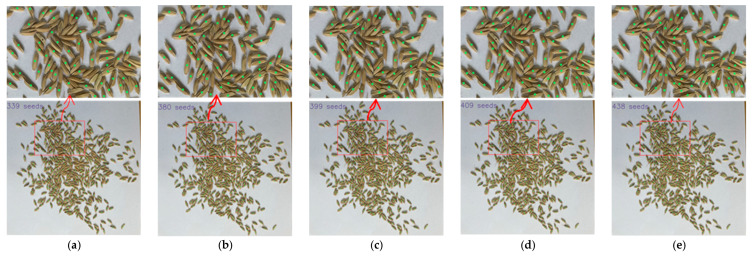
Comparison of detecting and counting results of YOLOv5s with different modules for severely adhesive rice grains. (**a**) Detecting and counting results of YOLOv5; (**b**) detecting and counting results of YOLOv5 + transformer; (**c**) detecting and counting results of YOLOv5 + transformer + CA module; (**d**) detecting and counting results of YOLOv5s + transformer + CA module + 4 prediction heads; (**e**) detecting and counting results of TCLE–YOLO.

**Figure 12:**
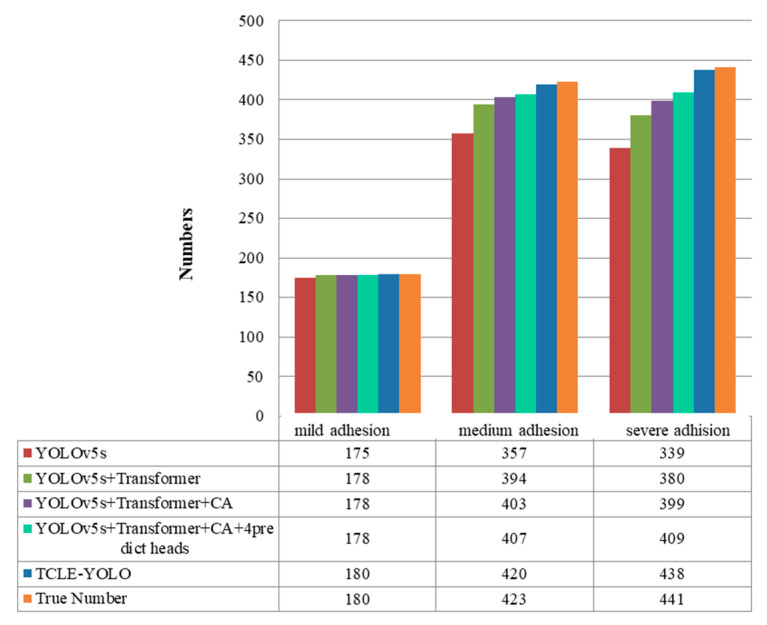
Counting results of YOLOv5s with different modules for the test dataset.

Figure 9, Figure 10 and Figure 11 show parts of the detection and counting results produced by YOLOv5s with different modules using the self-built data set. To show the results more clearly, image regions of the red boxes were zoomed and shown as the graphes where the arrows pointed. The detected grain target is represented by the center point of a predicted box corresponding to the target in the image. The number of center points was used to represent the number of rice grains in the image. It was found that the TCLE–YOLO model proposed in this study had better robustness and counting results, even when the rice grains showed severe adhesion. In order to illustrate the detection and counting performance of the model more intuitively, the detection results are counted so as to show the counting results, as is shown in Figure 12. As can be seen in Figure 12, the YOLOv5s with different modules can detect almost all mildly adhesive grains, but the TCLE–YOLO model produced an outstanding counting result for severely adhesive rice grains. In summary, combining a transformer, a CA module, four prediction heads, and EIoU loss yielded the best performance. This indicates the power of combining the different modules mentioned above, as these can play a central role in guiding accurate thousand-grain weight measurements.

### 3.3. Comparison of Different Detection Models

In order to verify the detection performance of the detection model proposed in this study, we compared the detection results of the proposed model to those of the other four detection models, namely Faster R-CNN [29], SSD [30], EfficientDet [31], and YOLOv7 [32]. In the comparison experiment, the same dataset and loss function mentioned above were used for the five detection models, and the evaluation indexes introduced in Section 3.1 were applied to examine the detection models. The detection performances of the different models are shown in Table 2.

Table 2 shows that the performance of the proposed model was better than that of the other four models for rice grain detection. Comparatively, the precision, recall, *mAP*@0.5, and *mAP*@0.5:0.9 of the proposed model were 99.20%, 99.10%, 99.20%, and 72.20% higher, respectively, than those of the Faster R-CNN model. Compared with Faster R-CNN, the precision and *mAP*@0.5:0.9 values of EfficientDet were slightly better, but its other two evaluation indicators were lower. SSD performed better than Faster R-CNN and EfficientDet, but the evaluation indicators, including *mAP*@0.5 and *mAP*@0.5:0.9, were still low. By comparison, YOLOv7 performed better in all the evaluation measures, i.e., its precision was approximately 8.77% higher than that of SSD, and its recall was approximately 9.65% higher. However, YOLOv7 was still not comparable to the proposed model, and it had lower FPS. The biggest difference was in *mAP*@0.5:0.9; the *mAP*@0.5:0.9 of the proposed model was 22.58% higher than that of YOLOv7. The experimental results indicate that the designs of the attention mechanism and detection head for small targets, as well as the introduction of EIoU loss in the model, elevated the model and enabled it to focus on features of interest and significant regions. During the training process, the model learned more details about rice grains of different degrees of adhesion, demonstrating its superiority in the task of rice grain detection compared with the other four models.

**Figure 13:**
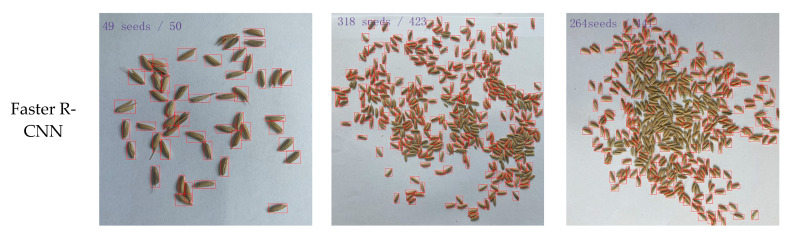

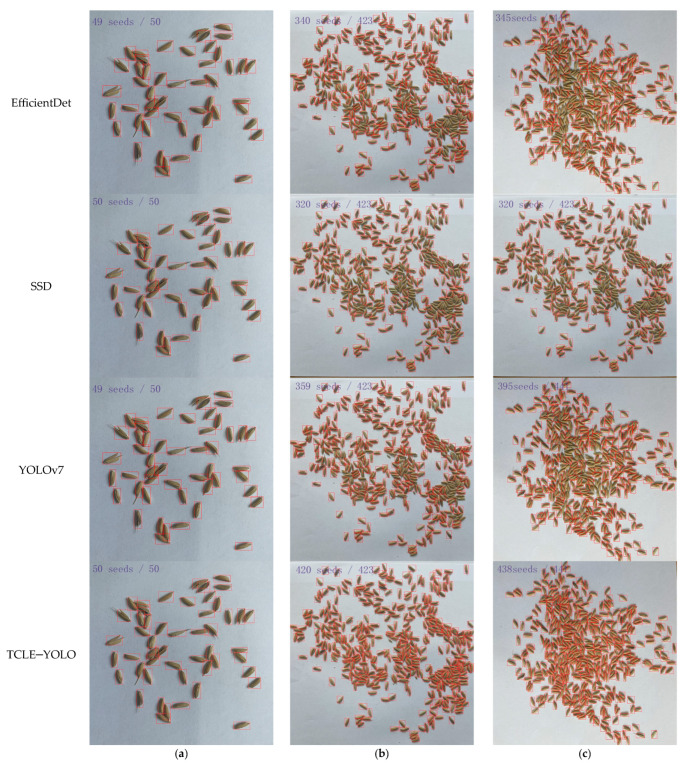
Detecting and counting results of different models. The images (**a**–**c**) were randomly selected from the test set.

In addition, several images were randomly selected from the test set for detailed comparisons, and the visual comparisons of the detection results using the five models are presented in Figure 13. All the images in the first, second, and third columns are, respectively, the final prediction results for mildly adhesive grains, moderately adhesive grains, and severely adhesive grains for each model. It was observed that the TCLE–YOLO model proposed in this study can accurately detect and count rice grains of different degrees of adhesion. This is because, in this model, a channel attention mechanism was introduced into the backbone module of YOLOv5, enabling it to learn more features about rice grains of different degrees of adhesion. The detection head, the design of which was based on a low-level, high-resolution feature map generated by the transformer encoder, can enhance the extraction of key features of detected targets, thus enabling the model to be more sensitive to heavily adhesive grains. The detection results of the Faster R-CNN, EfficientDet, SSD, and YOLOv7 models were, however, not as good as those of the proposed model, with especially poor adaptability to heavily adhesive grains. As Figure 13c shows, the Faster R-CNN, EfficientDet, SSD, and YOLOv7 models all incorrectly identified many rice grains as background. Moreover, the number of the predicted box of rice grains represents the number of rice grains in the image. From the figure, it can be seen that the four other models missed many detections of highly adhesive rice grains compared with the TCLE–YOLO model, the proposed model producing a result closer to the true number.

### 3.4. Discussion

The thousand-grain weight of rice grains has become an important indicator for estimating rice yields and evaluating cultivation measures, seed phenotypic analyses, variety breeding, imperfect rice grain sorting, etc., and it therefore necessitates the accurate detection and counting of grains. Because rice grains are small, low-resolution targets with high overall similarity in terms of color and morphological characteristics, and which possess different degrees of adhesion, the improved YOLOv5 model based on an attention mechanism and an additional detection head sensitive to small targets can increase detection accuracy and recall metrics. In the experiments we performed, there were still a few missed detections, and severe occlusion between rice grains resulted in undercounts. In the future, we will design an image acquisition platform that will include two processional cameras and an automatic motion device to make the acquisition of pictures easier and more accurate and solve the occlusion problem with multi-view imaging via binocular cameras. 

Although the experiments outlined above confirmed that the proposed model performed well when detecting and counting rice grains of different degrees of adhesion, we only carried out the experiments using a self-built dataset. Before it is truly implemented in practice, we should further improve the generalizability of the model and design a hardware platform for detecting and counting grains. The improved model will then be integrated into the designed device. Through the accurate detection and counting of rice grains, agricultural producers and rice breeders can estimate yields and evaluate cultivation measures and rice production management. Moreover, deep learning technology can help realize the automatic detection and counting of rice grains, which will improve real and reliable data support for phenotype measurements. In addition, the improved model can be applied to the detection and counting of other small seed cereals.

## 4. Conclusions

In this study, a detection model named TCLE–YOLO, which was based on an improved YOLOv5 model for rice grains, was presented. To reduce rice grain misidentification, especially for heavily adhesive rice grains, which are difficult to distinguish, an attention mechanism was embedded into the YOLOv5 and an additional detection head for small targets was designed. The model was trained, validated, and tested using a self-built dataset. The final test set scores were 99.20%, 99.10%, 99.20%, and 72.20%. Furthermore, compared with the Faster R-CNN, EfficientDet, SSD, and YOLOv7 models, the proposed TCLE–YOLO model had better detection and counting results for rice grains of different degrees of adhesion. The experiments, therefore, confirm that the proposed model performed well when detecting and counting rice grains of different degrees of adhesion. This provides objective support to applications such as thousand-grain weight measurements, rice breeding, and cultivation management. In the future, we will further improve the generalizability of the model and extend the application of the improved model to the detection and counting of other small seed cereals.

## Figures and Tables

**Table 1:** Comparison of evaluation indexes of YOLOv5s with different modules using the self-built dataset.

Model	*P* (%)	*R* (%)	mAP@0.5 (%)	mAP@0.5:0.9 (%)
YOLOv5	97.90	98.10	97.50	64.30
YOLOv5 + transformer	98.20	98.30	98.20	64.50
YOLOv5 + transformer + CA module	98.40	98.40	98.50	67.20
YOLOv5 + transformer + CA module + 4 prediction heads	99.10	99.00	99.00	71.20
YOLOv5 + transformer + CA module + 4 prediction heads + EIoU (TCLE–YOLO)	99.20	99.10	99.20	72.20

**Table 2:** Comparison of evaluation indexes of different models.

Model	*P* (%)	*R* (%)	mAP@0.5 (%)	mAP@0.5:0.9 (%)	*FPS*
Fast R-CNN	86.00	82.20	54.20	20.80	37
EfficientDet	93.23	67.70	50.72	24.70	42
SSD	90.10	90.20	64.30	25.00	37
YOLOv7	98.00	98.90	98.90	58.90	7
TCLE–YOLO	99.20	99.10	99.20	72.20	39

## Data Availability

Data are contained within the article.
